# Cold sintering of YBa_2_Cu_3_O_7−*δ*_

**DOI:** 10.1039/c9ra08744c

**Published:** 2019-12-11

**Authors:** James Cockburn, Rebecca Boston

**Affiliations:** Materials Science and Engineering, Sir Robert Hadfield Building, University of Sheffield Sheffield UK r.boston@sheffield.ac.uk

## Abstract

Cold sintering is a sintering technique which enables ceramic powders to be densified at greatly reduced temperatures compared to traditional solid state techniques, which often require temperatures in excess of 1000 °C. These temperatures often preclude the exploitation of size or orientational effects in ceramics as these are lost during heating. One such effect is the orientation of the crystallographic *c* axis in YBa_2_Cu_3_O_7−*δ*_ (YBCO) which can be controlled through applied pressure. This effect is of interest for increasing critical current density which is highly dependent on the orientation of the *a*–*b* (CuO_2_) planes within the ceramic. Using cold sintering, we demonstrate that dense YBCO can be created at 180 °C (*vs.* 1000 °C using solid state) and demonstrate that the likely sintering mechanism is mediated by the cracking which occurs in YBCO when exposed to water. In addition, the ceramics produced show and retain the orientational effect, representing a unique opportunity to study the effect on critical current density. We show that the intergranular critical current when the *a*–*b* planes are parallel to the applied field is around 15% higher than when perpendicular.

## Abstract 2

Cold sintered superconducting YBa_2_Cu_3_O_7−*δ*_ densified at 180 °C shows enhanced critical current densities by exploiting grain alignment created during pressing.

## Graphical abstract

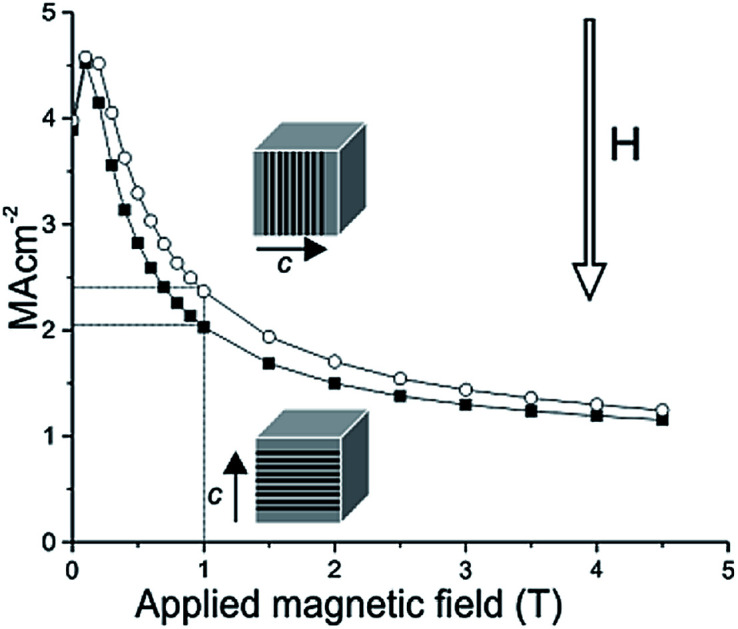

## Introduction

First created and tested in 1987,^1^ YBa_2_Cu_3_O_7−*δ*_ (YBCO) along with other members of the cuprate superconductor family^2^ are widely regarded as having re-ignited the field of superconductivity, by significantly lifting the superconducting transition temperature and forming the foundation for discovery of new superconducting materials.^3^ As a material, YBCO is relatively straightforward to make using solid state processing,^4^ however like many of the cuprate superconductors,^5^ the superconducting transition temperature is highly sensitive to oxygen non-stoichiometry,^6^ and so the high temperatures used to densify the ceramics mean that an oxygen anneal is often required to create the optimal oxygen content.^7^

Another feature of YBCO is that it displays preferential crystallographic orientation under applied pressure,^8^ which is potentially beneficial due to the high dependency of critical current on crystallographic orientation.^9^ The axes of crystallites align such that the *c*-axis is perpendicular to the applied force, an effect which is retained after the pressure is removed, however the usual subsequent high temperature sintering enables the crystallites to relax to create the usual distribution of crystal faces.

It is well known that superconductivity in YBCO (and other cuprates) originates in the CuO_2_ square planar sheets, which lie in the *a*–*b* planes.^10^ Correspondingly, these are weakly coupled in the *c*-direction, therefore creating the quasi-two-dimensional electron dynamics which are thought to govern superconductivity in the cuprates.^10,11^ If retention of the pressure effect could be achieved, it could be used to improve the critical current density, *J*_c_, of the materials created.^12^*J*_c_ can be increased through crystallographic alignment of grains within the bulk ceramic. Indeed, a crystallographic mismatch of only 7% can greatly reduce the critical current density as the CuO_2_ planes misalign creating phonon scattering sites,^9^ and so techniques to maximise crystallographic alignment are desirable. As a result, much of the recent YBCO research has been focused on manufacturing methods to create either uniaxed crystals or tapes which create better grain alignment such as MOD/RABiTs.^13^ If, however, the orientation observed in pressed YBCO could be preserved after sintering, this could act as a means to improve critical current in a controlled manner, however this requires a shift in sintering technique which does not allow the oriented grains to relax.

Sintering technologies have remained largely unchanged for thousands of years. In 2016, however, Guo *et al.* described a new mechanism, the cold sintering process,^14^ which enabled densification of ceramics at unprecedented low temperatures without significant detriment to functional properties. The majority of the initial materials were highly soluble, such as Li_2_MoO_4_, utilising water as a transient solvent to hydrate the surface of powder particles.^14^ Under pressure these particles flow and compact, with the hydrated phase being re-crystallised onto the surface of the powder phase to back-fill all of the voids between particles.^15^ This results in a fully dense ceramic at temperatures as low as 120 °C, the elevated temperature only needing to be high enough to evaporate off any transient solvent phase. Since 2016 this has been expanded into a wide range of other oxides including ternary and above,^16^ employing a range of chemical techniques in order to overcome issues with insoluble^17^ or incongruently soluble materials.^18^ Of all of the materials cold sintered to date, high temperature superconductors such as YBCO have not yet been investigated.

It has been well established that YBCO loses its superconducting properties in the presence of water.^19^ When exposed to moisture, YBCO has been shown to decompose into BaCO_3_, CuO and Y(OH)_3_.^20^ Interestingly, although insoluble in water, bulk YBCO samples have been shown to form an amorphous surface layer in the presence of water, similar to that in as is observed during cold sintering of soluble materials.^21^ This layer may well facilitate the cold sintering process in YBCO at greatly reduced temperatures compared to solid state sintering. Given the known pressure-induced orientational effect which should be retained in the ceramic this represents the opportunity to study its effect on the superconducting properties in dense ceramic pieces. In addition, smaller particle size generally results in higher critical current densities, and so the ability to control and minimise particle size during sintering is also desirable; high sintering temperatures generally results in grain growth.

Herein we describe the application of cold sintering to YBCO solid state powders to create fully dense ceramics at 180 °C, and compare the superconducting properties with samples sintered using conventional high temperature techniques.

## Experimental

### Synthesis

YBa_2_Cu_3_O_7−*δ*_ powder was synthesised using standard solid state processes. Y_2_O_3_, BaCO_3_ and CuO powders (Sigma Aldrich, UK) were dried, batched, and milled, followed by calcination at 950 °C for a total of 24 h in air with intermittent re-grinding.

### Sintering

Solid state sintered samples were pelletised using a uniaxial press and sintered in air at 1000 °C for 8 h. Cold sintered samples were prepared by hand grinding the calcined powder in a pestle and mortar with a 1 : 1 ratio by mass of water until the powder appeared dry, approximately 10 minutes for a 0.3 g sample. The powder was then transferred to a die and pressed at 600 MPa for 10 minutes at room temperature. The temperature of the press was then increased to 180 °C at a rate of 7 °C min^−1^ and held at temperature for 1 hour. Following this, the die was allowed to cool with pressure still applied.

### Characterisation

X-ray diffraction was performed using Bruker D2 and Malvern Panalytical X'Pert^3^ diffractometers using Cu K_α_ radiation, with surfaces polished before measurements were conducted.

Density measurements were made using the Archimedes technique in water, with the surfaces of the ceramics carefully dried and polished after testing to remove layers which may have reacted with water.

Samples for transmission electron microscopy (TEM) were prepared by manually thinning a section of sintered ceramic followed by ion beam milling using at Gatan PIPS II. TEM was conducted using a JEOL JEM-2010 in bright field mode. Energy dispersive spectroscopy (EDS) was used to identify the relative quantities of each element present using an Oxford Instruments EDS detector.

Solid state sintered samples were thermally etched at 90% of sintering temperature for 30 minutes to expose grain boundaries before being prepared for scanning electron microscopy (SEM). Cold sintered samples were not thermally etched as this would greatly change the thermal history of the samples, instead they were cut to size and polished. All SEM samples were affixed to double sided carbon tape and sputtered with approx. 15 nm of gold before imaging. Imaging was performed using a Philips Inspect F SEM.

Magnetometry data were collected using a Quantum Design Superconducting Quantum Interference Device (SQUID) magnetometer. Magnetisation *vs.* temperature measurements were conducted between 10 and 150 K with an applied field of 100 Oe. Magnetisation *vs.* field measurements were collected between −4.5 and 4.5 T at 10, 50 and 77 K, with a virgin loop taken to create the initial hysteresis. Samples were mounted such that the direction of *c*-axis overexpression was known. The critical current *J*_c_ was calculated using the Bean model,^22^ with particle sizes estimated from a statistically significant sample of particles (>500) from SEM, taking the long axis, and density used as found using the Archimedes technique.

## Results and discussion

**Fig. 1:**
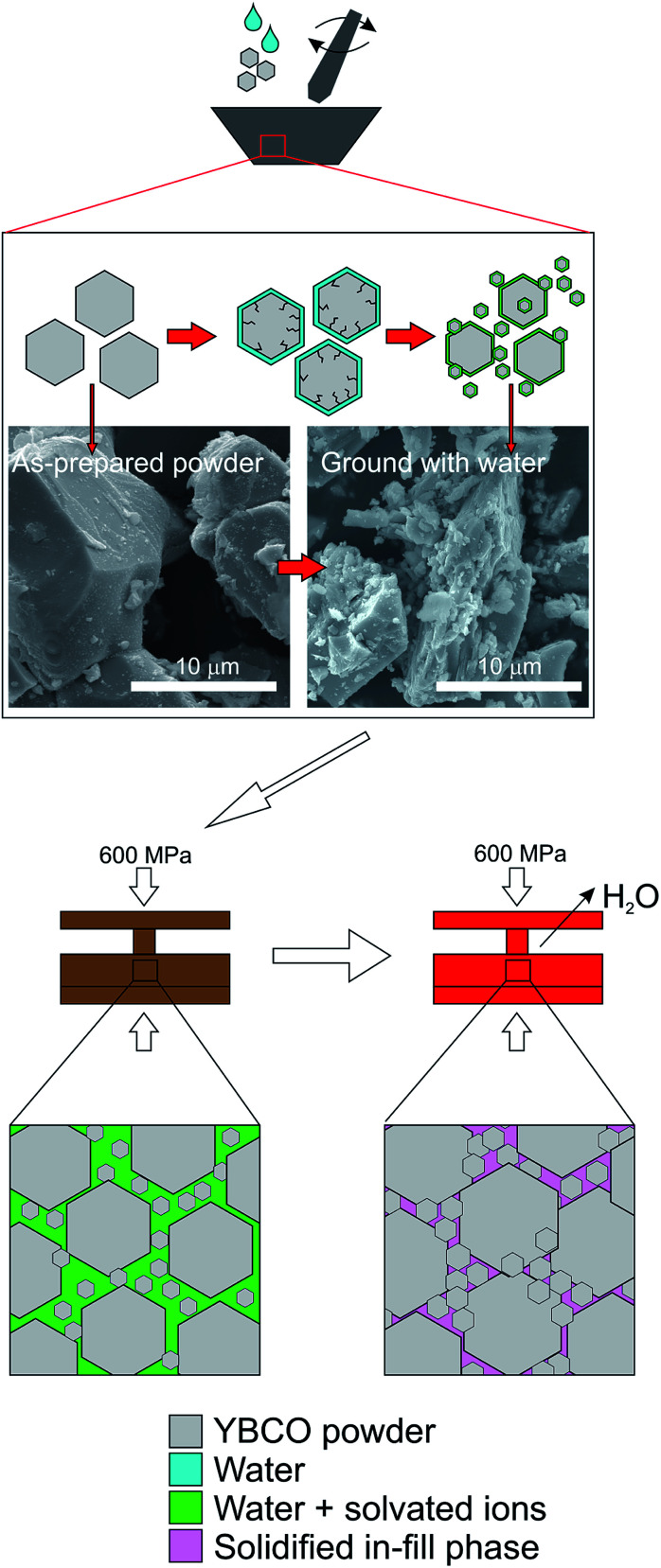
Schematic of the proposed sintering mechanism. YBCO powder ground with water undergoes cracking resulting in a reduction in “bulk” powder size, and the creation of numerous smaller particles as demonstrated in the SEM micrographs. All particles are hydrated and this also causes egress of Y, Ba and Cu ions from the particles. This solution phase enables flow of particles past one another during room temperature pressing creating a high green density. During heating inside the die, the last of the water is evaporated causing re-deposition, and/or crystallisation and/or precipitation of the solution phase, resulting in a fully dense ceramic.

Conventional water-based cold sintering was used to successfully produce fully dense YBCO ceramics with densities of 87 ± 1% of theoretical *vs.* 89 ± 1% using traditional solid state sintering at 1000 °C. The cold sintering result is perhaps surprising as YBCO is insoluble in water, however the known partial decomposition is likely to be responsible. We therefore postulate the following mechanism (Fig. 1) for cold sintering of YBCO: the addition of water, followed by grinding, causes the breakdown of some of the starting powder particles into smaller particles surrounded by an amorphous layer composed of the three metal ions. This is attributed to the cracking process known to occur when YBCO is exposed to water,^19,20^ the results of which can be observed in the SEM images in Fig. 1. SEM before and after grinding with water show significant differences in particle size compared to the as-prepared powder, with an increase in the number of smaller grains present. A combination of a range of particle size and hydrated surfaces enables efficient packing as particles can flow past one another by capillary action during the room temperature hold. Remaining voids are then filled by the ion-rich hydrated phase which also acts to sinter grains together during the heating step. Upon heating, any remaining water is evaporated, creating a dense ceramic by in-filling spaces between grains. This is distinct from standard solid state sintering where surface energy is minimised by decreasing surface area (*i.e.* grain growth).

**Fig. 2:**
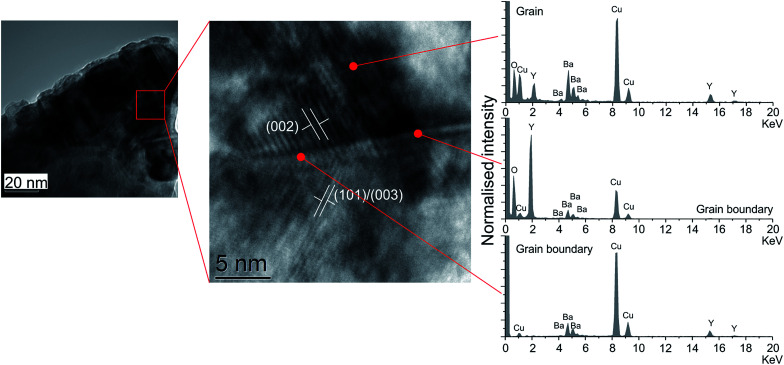
TEM micrograph of a section of a cold sintered ceramic, with a highlighted high resolution area with (*hkl*) planes shown for two grains. EDS spectra are given for the bulk grain and two regions within the grain boundary as indicated. The grain shows a typical spectrum for YBCO, in contrast to two off stoichiometry regions, one relatively higher in yttrium, and one relatively higher in copper demonstrating compositional inhomogeneity across the grain boundary.

In order to prove this hypothesis and to investigate the nature of the grain boundaries, transmission electron microscopy (TEM) and energy dispersive spectroscopy (EDS) was conducted to examine the composition of the grain boundaries *vs.* the grain. Fig. 2 shows bright field images and corresponding EDS spectra of a region of cold sintered ceramic.

A higher resolution area containing a grain boundary is also highlighted. Interference fringes visible in the grains have been used to identify the corresponding (*hkl*) lattice planes, and the corresponding EDS spectra shown for different regions. The area within the grain has a relative distribution of Y, Ba and Cu as would be anticipated from a 1 : 2 : 3 stoichiometry. This is in contrast to the grain boundary. Two spectra are shown, one which has a relatively higher quantity of copper, the other of yttrium. Both of the grain boundary spectra contain similar relative quantities of barium ions, indicating that the barium tends to distribute itself isotropically throughout the grain boundaries.

The EDS shows that the forming grain boundary phase is not only non-stoichiometric with respect to the starting powder, and that the grain boundaries are compositionally inconsistent across the sample. The known decomposition of YBCO upon contact with water^19,21^ is likely to be providing a stoichiometric mixture of ions to form the grain boundary phase, however during cold sintering this appears to be segregating to form distinct regions rich in each ion. This is likely to have the effect of reducing the total superconducting volume fraction in the cold sintered samples.

**Fig. 3:**
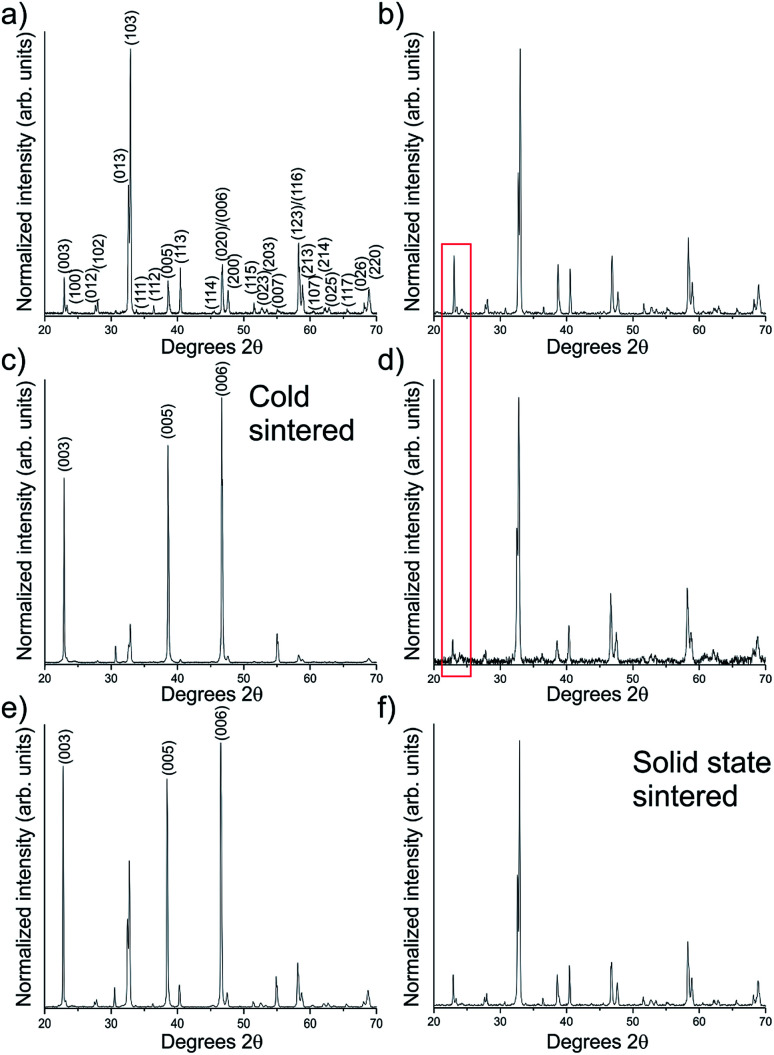
X-ray diffraction patterns for (a) the as-prepared YBCO powder, showing normal relative intensities. (b) Shows the same powder ground with water in preparation for cold sintering. Some over expression of the (00*l*) faces is observed. (c) Pattern obtained from the top surface of a cold sintered ceramic showing significant (00*l*) plane overexpression, in contrast to the inner surface of the same ceramic in (d) which shows a corresponding under-expression of the (00*l*) planes. Red box highlights the difference in relative intensities of the (003) reflection in the powder *vs.* the internal surface of the cold sintered pellet, indicating a relative decrease. The (00*l*) overexpression is also observed in (e), which is a sample uniaxially pressed at room temperature ready for standard solid state sintering, which also shows some overexpression in the *c*-direction, in contrast to the same sample after sintering at 1000 °C in (f) which has returned to its isotropically axed state.

X-ray diffraction (XRD, Fig. 3), was used to confirm the phase present. Fig. 3a and b display the XRD patterns of the as-prepared solid state powder and the same powder ground with water in preparation for cold sintering respectively, with no compositional differences observed. If decomposition into other materials is occurring, it is at a level below the detection limit of the XRD used (<4 wt%). One point of note is the slight increase in the (00*l*) peaks as indicated in Fig. 3b, as a result of the pressure applied during grinding.

This (00*l*) overexpression is significantly increased after cold sintering, as displayed in Fig. 3c, which shows the top surface of a cold sintered ceramic *i.e.* the surface orthogonal to the direction of applied pressure. This stands in contrast to the inner surface of the ceramic which shows a corresponding reduction in the (00*l*) faces *vs.* the normal relative intensities Fig. 3d and red box. The orientational effect is also observed, albeit to a lesser extent, in the solid state sintered samples after they have been uniaxially pressed but before heating (Fig. 3e). This indicates that the overexpression of (00*l*) faces is caused by the pressure applied in the die. The high temperature sintering step enables relaxation of this effect, returning the crystallites to an isotropically axed state, Fig. 3f, which shows the same pattern as the starting powder, Fig. 3a.

**Fig. 4:**
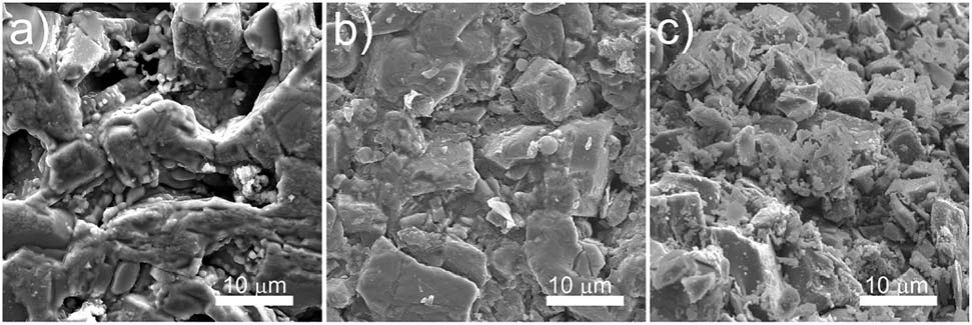
SEM micrographs of (a) a thermally etched solid state sintered ceramic, (b) a polished, cold sintered ceramic and (c) a fracture surface of the cold sintered sample.

Morphology and physical structure of the ceramics was examined using scanning electron microscopy (SEM), Fig. 4.

The micrograph in Fig. 4a indicates that the thermally etched solid state sintered sample is porous in line with the measured density, and composed of large, partially melted particles indicative of some liquid phase sintering. The average particle size (long-axis) was measured to be 3.6 ± 1.4 μm. This is in contrast to Fig. 4b and c which show the native surface and fracture surface respectively of a cold sintered ceramic (the higher temperatures required by thermal etching would fundamentally alter the grain structure and so was not used in the cold sintered cases). Here the grain size is measured to be smaller: 1.5 ± 1.2 μm due to the water-induced cracking and lack of grain growth during preparation. The particles in the sample are well packed, again in line with the measured density.

**Fig. 5:**
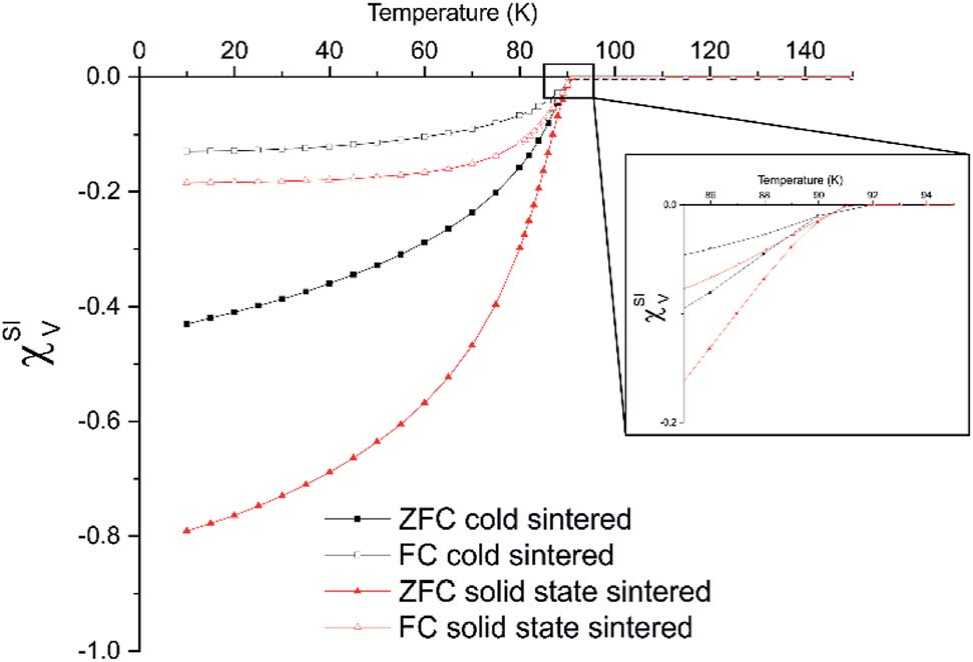
Magnetisation *vs.* temperature SQUID magnetometry of cold and solid state sintered ceramics under both field and zero field cooled conditions. Data is given in terms of the dimensionless volume susceptibility.

Superconducting quantum interference device (SQUID) magnetometry was used to determine superconducting transition temperature and volume susceptibility using magnetisation *vs.* temperature sweeps (Fig. 5). For both the standard sintered (Fig. 5a) and cold sintered (Fig. 5b), the onset superconducting transition temperature was 91 K, indicating a good level of oxygenation and a nominal formula of YBa_2_Cu_3_O_6.9_.^6^

The volume susceptibility also gives an approximation of the superconducting volume of the sample. The solid state sintered samples show a superconducting volume of around 80% *vs.* around 45% in the samples created using cold sintering. There are two causes of this. Firstly, the smaller grain size of the cold sintered samples creates a greater volume of field ingress arising from the penetration of the magnetic field (determined by the London penetration depth).^23^ Secondly there will be a small volume of non-superconducting material present in the in-fill phases between grains, as observed in the TEM (Fig. 2). Interestingly, the field cooled measurements are similar in across all samples. This also indicates that any non-superconducting phase in the cold sintered sample is likely to be located in the grain boundaries rather than regions within the grains themselves.

**Fig. 6:**
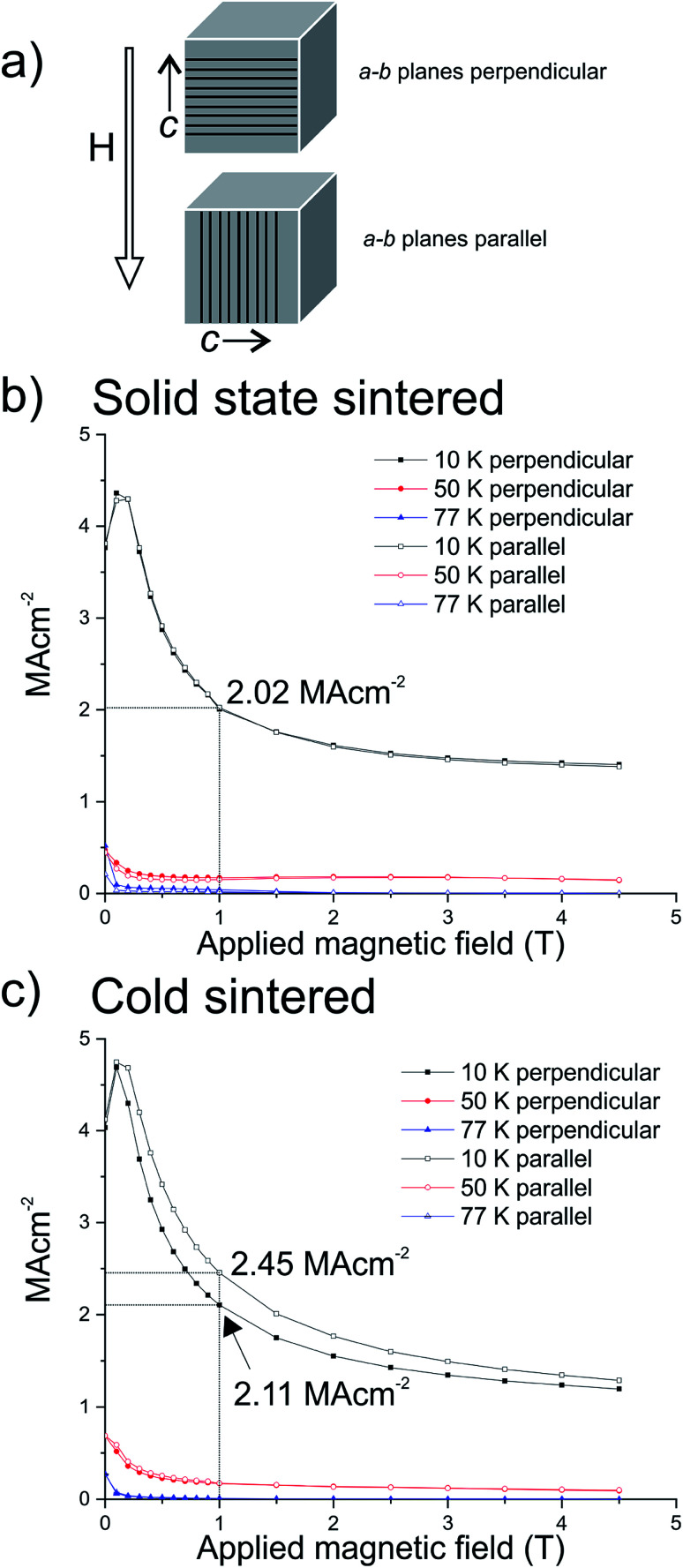
(a) Schematic of cold sintered samples to show orientation of *a*–*b* planes parallel and perpendicular with respect to the applied magnetic field inside the SQUID. (b) Representative critical current densities calculated using the Bean model at 10 K, 50 K, and 77 K of the solid state sintered samples oriented with *a*–*b* planes perpendicular and parallel to the applied magnetic field. No significant difference between the orientations is observed as would be anticipated in an isotropically axed ceramic. (c) Critical current densities for a cold sintered sample with the *a*–*b* planes perpendicular and parallel to the applied magnetic field, demonstrating that as hypothesised, the sample oriented with the *a*–*b* planes parallel to the applied field is able to carry a higher critical current than perpendicular due to the crystallographic orientation within the sample.

Despite the slightly lower superconducting volume, cold sintered samples represent an opportunity to study the pressure-induced orientational effect, without the alignment being lost during sintering as would normally occur in solid state sintered ceramics, Fig. 6. Particle sizes from SEM were used in conjunction with magnetisation *vs.* field SQUID magnetometry at fixed temperatures to calculate intergranular critical current density using the Bean model.^22^ In both cases the particle size is widely distributed, with larger grains surrounded by much smaller particles which will have an impact on the calculation of *J*_c_. The value of particle size used for the Bean model takes the average long axis of a statistically significant number of these particles from SEM (*e.g.*Fig. 4). As the ceramics have a wide variation in particle size the averages used in the Bean model calculations are correspondingly skewed to the smaller end of the distribution as the smaller particles are more numerous in each case.

Using XRD as a guide, samples were orientated inside the SQUID with the *a*–*b* planes parallel or perpendicular to the applied magnetic field; a schematic of these orientations is shown in Fig. 6a. We hypothesise that the isotropically axed solid state samples should show the same size of critical current regardless of orientation with respect to the applied field, whereas the cold sintered samples should show a difference. This arises from the retention of pressure-induced preferred orientation shown in the cold sintered samples: if the overexpressed *a*–*b* CuO_2_ planes are parallel to the applied field, then a greater amount of supercurrent should flow, *versus* when the overexpressed *a*–*b* planes are perpendicular, as a supercurrent flowing in the *c*-direction has a higher energy cost.^12^

Fig. 6b indicates that, as predicted, there is no difference between solid state sintered ceramics oriented parallel or perpendicular to the applied magnetic field with respect to the direction of applied pressure, as would be expected following relaxation of the preferential crystallographic orientation during sintering. The value of the critical current at 10 K and 1 T of applied field was found to be 1.9 MA cm^−2^ regardless of the orientation of the sample.

In contrast, the cold sintered ceramics oriented perpendicular and parallel to the applied magnetic field show differences in the size of the critical current, Fig. 6c. The cold sintered ceramic with the over-expressed superconducting *a*–*b* planes parallel to the applied field shows a markedly higher value of critical current 2.45 MA cm^−2^ as opposed to 2.11 MA cm^−2^ in the sample with the *a*–*b* planes perpendicular to the applied field (both at 10 K and 1 T of applied field). It should be noted also that both of these values are higher than the solid state sintered ceramics, predominantly due to the smaller particle size of the cold sintered samples, and higher still than might be expected due to the relatively smaller superconducting volume.

## Conclusions

We have presented cold sintering as a method by which ceramic YCBO samples with preferential crystallographic orientation can be fabricated. Cold sintering presents a new route to fabrication which both uses less energy than conventional sintering and a means by which the superconducting properties can be improved. Cold sintered samples show improved critical current densities over their solid state sintered equivalents, and with the preferential orientation parallel to the applied field, further improvement of the critical current can be achieved. This is likely to be further improved in combination with smaller particle size. We propose that the mechanism by which cold sintering is occurring in YBCO is due to partial decomposition of the surface of the starting powder creating an infill phase, indicated by the field cooled measurements to be non-superconducting. This infill phase creates thin grain boundary regions with variable composition, but has apparently little impact on the superconducting properties, and could be further investigated by for example AC impedance spectroscopy. The measured intergranular critical current in the cold sintered samples was higher than the solid state sintered samples, driven by a combination of retained orientation of the crystallites, and a smaller particle size. This may have useful application in industrial processing of YBCO tapes.

## Conflicts of interest

There are no conflicts to declare.

## Supplementary Material
